# Fusion in the ETS gene family and prostate cancer

**DOI:** 10.1038/sj.bjc.6604558

**Published:** 2008-09-09

**Authors:** S A Narod, A Seth, R Nam

**Affiliations:** 1 Department of Medicine, Womens College Research Institute, University of Toronto, ON, Canada; 2 Department of Pathology, Sunnybrook Research Institute, University of Toronto, ON, Canada; 3 Divisions of Urology and Surgical Oncology, Sunnybrook Health Sciences Centre, Toronto, ON, Canada

**Keywords:** prostate cancer, translocation, fusion, ERG, ETS, TMPRSS2

## Abstract

It has recently been shown that the majority of prostate cancers harbour a chromosomal rearrangement that fuses the gene for an androgen-regulated prostate-specific serine protease, *TMPRSS2*, with a member of the ETS family of transcription factors, most commonly *ERG*. These are among the most common genetic alterations in any human solid tumour. This knowledge may provide us with clues to prostate carcinogenesis, and may lead to the development of important molecular-based biomarkers for patients with localised prostate cancer. The most common variant is fusion between the 5′-untranslated region of *TMPRSS2* and the 3′ region of *ERG*. However, over 20 other fusion variants have now been described (involving over 10 different genes) and the number of variants continues to grow. Fusion products can be identified by several techniques, including FISH, RT–PCR, and expression profiling using exon arrays. The protein products associated with the fusion transcripts have not been characterised, and the phenotypic expression of the various products of gene fusion on prostate cancer histology, or on the clinical course of cancer, are not yet understood. Several early cohort studies suggest that the presence of the *TMPRSS2:ERG* fusion product is associated with relatively poor cancer-specific survival. Studies that examine how individual variants and their associated phenotypes affect prostate cancer presentation and progression are required.

## Fusion of Tmprss2 and Ets Transcription Factor Genes

In 2005, Tomlins *et al* identified and described a small number of fusion transcripts, specific to prostate cancer, that were the consequence of a chromosomal rearrangement involving two genes. One gene, *TMPRSS2* (androgen-regulated trans-membrane protease, serine 2), encodes for a serine protease that is secreted by prostate epithelial cells in response to androgen exposure (Afar *et al*, 2001). *TMPRSS2* was fused to either *ERG* or *ETV1*, two members of the ETS family of oncogenes (Tomlins *et al*, 2005). It had earlier been reported that the *ERG* gene was the most commonly overexpressed proto-oncogene in prostate cancer (present in about 72% of cases of prostate cancer) (Petrovics *et al*, 2005), and Tomlins *et al* now proposed a mechanism to explain the overexpression. In this landmark paper, they found that both intra-chromosomal and inter-chromosomal genetic rearrangements led to the creation of a fusion transcript called *TMPRSS2–ETS*. ETS is a family of transcriptional activators and inhibitors. Their activity is regulated by phosphorylation and protein–protein interactions (Seth and Watson, 2005). *ERG*, *ETV1*, *ETV4*, and *ETV5* are members of the *ETS* family. The *ERG* gene is located on chromosome 21q, *ETV1* is located on chromosome 7p, ETV4 on chromosome 17q, and *ETV5* on chromosome 3q. Using a novel and powerful bioinformatic technique, they first determined that either *ETV1* or *ERG* (but not both) was commonly overexpressed in prostate cancer cells. Furthermore, overexpression did not usually include all *ERG* exons – exons 4–7 were overexpressed much more commonly than were exons 1 and 2. This intriguing observation suggested to them that the gene had somehow been broken – and by sequencing the cDNA products, they confirmed that the 5′ part of the *ERG* gene had been replaced by the sequence derived from the *TMPRSS2* gene. They were able to verify the rearrangement at the DNA level with FISH techniques. Using expression arrays derived from prostate cancer specimens, they confirmed that one of the two *ETS* oncogenes was overexpressed in 57% of 167 prostate cancers. They concluded by showing that, in cells which carried either fusion product, expression of the *ERG* oncogene sequences was under the control of androgen. For example, VCaP cells, which harbour the fusion product *TMPRSS2:ERG*, expressed *ERG* transcript at a level 2000-fold greater than LNCAP cells, which do not harbour a fusion product. Because *TMPRSS2* is regulated in the prostate by androgens, it was proposed that this gene rearrangement could be a mechanism whereby the *ETV1* or *ERG* oncogenes were overexpressed, leading to prostate cancer.

The *TMPRSS2* and *ERG* genes are about 3 megabases (mB) apart on chromosome 21. In about two-thirds of cases, fusion is the result of the deletion of the intervening DNA sequence, but fusion may also occur by a more complex rearrangement, such as a translocation (Yoshimoto *et al*, 2006; Tu *et al*, 2007). The most common fusion is between the 5′-untranslated region of *TMPRSS2* and *ERG* (Perner *et al*, 2006; Yoshimoto *et al*, 2006; Tu *et al*, 2007; Mehra *et al*, 2007b). The specific points of DNA breakage, and the exons retained in the fusion product, differ between patients. Over 20 *TMPRSS2:ERG* variants have now been described (Tomlins *et al*, 2005, 2006; Clark *et al*, 2007; Liu *et al*, 2007). A nomenclature has been proposed to describe the variant transcripts, on the basis which exons of the genes are involved (Clark *et al*, 2007). Most variants are the result of a recombination between either exon 1 or exon 2 of *TMPRSSR2*, and exon 4 of *ERG* (although exons 2–5 may be involved). The most commonly reported fusion transcript is between exon 1 of *TMPRSS2* and exon 4 of *ERG* (Clark *et al*, 2007). This particular rearrangement is designated as T1/E4 according to the nomenclature proposed by Clark *et al* (2007). This transcript was originally described by Tomlins *et al* (2005), and in some studies, accounts for up to 86% of all reported fusions (Wang *et al*, 2006). Most patients who have been included in clinical and pathologic studies so far carry a variant involving these two genes (Wang *et al*, 2006; Demichelis *et al*, 2007; Lapointe *et al*, 2007; Nam *et al*, 2007b; Attard *et al*, 2008).

The number of genes and the variants involved in fusion transcripts continues to grow. Recently, it was found that other members of the *ETS* gene family (*ETV4* and *ETV5*) are involved in a small proportion of prostate cancer cases (Tomlins *et al*, 2006; Helgeson *et al*, 2008). New partners have also been identified on the 5′ side of the translocation. Lapointe *et al* (2007) identified a fusion product derived from a variant isoform of *TMPRSS2*, which mapped 4 kb upstream of the more common start site (Lapointe *et al*, 2007). One in 10 of the 63 prostate cancer cases in their series expressed this unique isoform. Tomlins *et al* (2007) and Helgeson *et al* (2008) implicated other 5′ fusion partners for ETV1, including *SLC5A3*, *HERV-K22q11.23*, *C15orf21*, and *HNRPA2B1*. *SLC5A3* appears to be capable of fusion to *ETV5*, as well as to *ETV1*, but not to *ERG* (Tomlins *et al*, 2007; Helgeson *et al*, 2008). Hermans *et al* (2008) identified two additional fusion partners of *ETV4* (kallikrein 2 (KLK2) and calcium-activated nucleotidase 1 (*CANT1*)) (Hermans *et al*, 2008).

Considering the new reports, it appears that a member of the ETS family is overexpressed in the majority of prostate cancers and that there may be mechanisms for overexpression other than through gene fusion. In the original paper by Petrovics *et al* (2005) a high proportion of cancers overexpressed *ERG*, but the underlying genetic mechanism was not determined. Cai *et al* (2007) reported that *ETV1* was overexpressed in the majority of prostate cancers, but that only the minority of these had evidence of a translocation (Cai *et al*, 2007). Hermans *et al* (2006) studied 11 prostate cancer xenografts, some of which exhibited androgen-dependent growth. In the androgen-dependent cases, the *TMPRSS2:ERG* fusion transcript was present, and overexpression of the *ERG* gene was associated with the expression of androgen receptor and PSA. Some androgen-independent cancers were also found to contain the *TMPRSS2:ERG* fusion transcript, but lacked androgen receptor – it is believed that these tumours have passed through an androgen-dependent phase. They also found two cases of advanced androgen-independent prostate cancer in which other members of the *ETS* family (specifically *FlI-1* and *ETV4*) were overexpressed due to a mechanism other than gene fusion. Of the 5′ fusion partners identified by Tomlins *et al* (2007), three (*TMPRSS2*, *SLC5A3*, and *HERV-K22q11.23*) appear to be androgen-responsive and two (*C15orf21* and *HNRPA2B1*) appear to result in constitutive overexpression of *ETV1* in the absence of androgen stimulation. In the future, clinical studies should distinguish the course of the disease in cases of cancer with different fusion proteins and the response to androgen ablation treatment.

## Characterisation of Tmprss2erg Protein

The consequence of the most common gene fusion is to generate a hybrid transcript that attaches the prostate-specific promoter sequence of the *TMPRSS2* gene to the ERG oncogene open reading frame (ORF). The proteins sequences have been predicted from the sequence of the fusion ORFs. Clark *et al* (2007) studied cDNAs prepared from *ERG* mRNAs isolated from 26 prostate cancers. They reported that of the 14 different fusion transcripts identified from the cDNA sequence, 5 would be predicted to generate premature stop codons and would be unlikely to encode for a functional ERG protein. In most cases, no amino acid sequence derived from TMPRSS2 is integrated in the hybrid ORF, and therefore a fusion protein is not created. In the case of the T1/E2 *TMPRSS2:ERG* variant, the full-length ERG protein is translated; the other fusion transcripts encode for a truncated protein (Clark *et al*, 2007).

## Prevalence of Fusion Product Among Unselected Prostate Cancer Cases

The presence of a gene fusion product can be determined by measuring the level of RNA expression using RT–PCR, by using FISH (which probes for inappropriate juxtaposition of non-adjacent sequences or the ‘break apart’ of a single gene to different chromosome locations), or by observing imbalance in the expression of individual exons using array technology. The prevalence rate of fusions among cancer patients depends on the assay used, the volume of the cancer, the number of cancer foci studied, and the number of fusion variants included in the screening panel. The question of prevalence is also complicated by the observation that a single cancer may have different foci that harbour rearrangements involving different genes, or no rearrangment at all (Clark *et al*, 2007). The field is evolving, but on the whole, these data suggest that 70% or more of all prostate cancers harbour a fusion product (Hermans *et al*, 2006; Perner *et al*, 2006; Soller *et al*, 2006; Rajput *et al*, 2007; Tu *et al*, 2007; Nam *et al*, 2007a); individual estimates vary from 15% (Demichelis *et al*, 2007) to 78% (Soller *et al*, 2006). As the number of variant species continue to increase and as techniques for measuring them become increasingly diverse and more sensitive, we are likely to find that an even greater proportion of prostate cancer specimens contain one or more fusion variants.

Prostate cancer is often a multifocal disease. Mehra *et al* (2007a) examined gene fusion status within different tumour foci from 43 patients with multifocal cancers, embedded in a tissue microarray (Mehra *et al*, 2007a). Of these patients, 70% had cancers that exhibited a fusion product, but in many cases, different foci from the same tumour carried different fusion products. Surprisingly, 70% of the cases with fusion were discordant for the specific fusion products. When the largest focus of cancer from each patient was identified, 83% of samples had a rearrangement. A similar result was obtained by Barry *et al* (2007), who studied 32 cases of multifocal prostate cancer. Of these cases, 41% exhibited heterogeneity (Barry *et al*, 2007). Recently, Clark *et al* (2007) also found that different cancer foci from a single patient harboured different fusion protiens. In some cases, both *ERG* and *ETV1* were involved in different foci of cancer from the same patient. Thus, heterogeneity of *TMPRSS2:ERG* gene fusion suggest that the different foci of cancer that arise within a multifocal prostate cancer probably have different origins and represent different malignant clones – this observation also implies that the clinical interpretation of this biomarker is more complex than was originally thought.

## Pathological Characteristics

Despite the lack of characterisation of the individual proteins, the phenotypic features of the *TMPRSS2:ERG*-associated cancers, as a class, have been described. Mosquera *et al* (2007) studied 120 cases of prostate cancer with the fusion gene, identified from a total of 253 cases (Mosquera *et al*, 2007). Five histologic features were associated with the presence of gene fusion: the presence of blue-tinged mucin, a cribriform growth pattern, macronucleoli, intraductal tumour spread, and signet-ring cell features – these features are also associated with an aggressive clinical course of prostate cancer – but neither Gleason grade nor stage was significantly associated with the presence of the fusion gene. Several other studies have compared the characteristic of prostate cancers with and without gene fusion in terms of grade and stage, and PSA level (Perner *et al*, 2006; Wang *et al*, 2006; Lapointe *et al*, 2007; Rajput *et al*, 2007; Tu *et al*, 2007). Results to date have been inconsistent, but most studies suggest that the presence of the fusion protein is not correlated with other markers of risk. Rajput *et al* (2007) found that fusions were less common in low-grade (Gleason 2) prostate cancers (1 of 17; 7%) than in moderate grade (Gleason 3–5) cancers (35 of 86; 41%; *P*-value for difference=0.02). Wang *et al* (2006) suggested that the T2/E4 variant might be associated with more aggressive disease than other fusion transcripts. This might be the case if the abundance of the oncogene transcript varied between cells with different fusion species, but this hypothesis is not confirmed. *TMPRSS2:ERG*-associated cancers have also been evaluated from the point of view of gene co-expression. Iljin *et al* have reviewed expression data derived from 410 different prostate tissue samples. They found that the most common gene that was co-expressed with ERG was histone deacetylase 1 (*HDAC 1*) and that, in fact, all the ERG-positive prostate cancers in that series were also strongly positive for *HDAC* (Iljin *et al*, 2006). This observation suggested to them that anti-HDAC therapies might have a potential therapeutic application for this class of prostate tumours (Li *et al*, 2005).

## Clinical Significance of Tmprss2:erg Gene Fusion

To date, there are three clearly established prognostic factors for men with localised prostate cancer: histologic grade (measured by the Gleason scoring system, tumour stage, and PSA level at diagnosis. Men with tumours of higher grade (Gleason 8–10), stage (T3–T4), or PSA level (e.g., >20 ng ml^−1^) experience relatively high rates of progression to metastasis, when compared with men with tumours of lower grade, local stage, or low PSA level. A biomarker will be clinically useful if it allows one to select treatments for individuals; that is if it helps identify subgroups of patients who will, or who will not, benefit from a specific treatment. A patient may not benefit from a treatment because survival is excellent in the absence of treatment, or because the rate of progression is high despite treatment.

In an early study, Petrovics *et al* (2005) found that *ERG* sequences were overexpressed in prostate cancer cells relative to adjacent benign prostate cells (at a level of twofold or greater) in approximately 80% of prostate cancers. In this study, patients with the highest level of expression in their cancer cells (relative to benign tissues) had the best prognosis, and the difference in survival between the groups was highly significant.

The clinical significance of the presence of the *TMPRSS2:ERG* gene fusion product on prostate cancer presentation and progression is not fully understood, but studies to date suggest that this may be a biomarker of risk. The results of the various studies are inconsistent, possibly because different study designs, different biomarkers, and different end points are used. In general, the case series (Lapointe *et al*, 2007; Rajput *et al*, 2007; Tu *et al*, 2007; Attard *et al*, 2008) and case–control studies (Perner *et al*, 2006; Wang *et al*, 2006) did not identify a significant prognostic effect. Using a case–control approach, Wang *et al* (2006) examined 119 patients for fusion status. They found a significant correlation with tumour stage, but not with early recurrence. Lapointe *et al* (2007) found modest correlations between stage and grade and the presence of the fusion product, but the sample size was small (*n*=63 cases) and neither association was statistically significant.

In contrast to the early study of *ERG* gene expression by Petrovics *et al* (2005), three recent cohort studies that evaluated the presence of the fusion protein *per se* on patient outcome have found the translocation to be an adverse prognostic factor. Two of these studies included patients undergoing watchful waiting. These authors sought to determine whether patients who do not harbour a fusion product might be candidates for watchful waiting. Currently, it is estimated that up to 30% prostate cancer patients lack the fusion protein, and therefore the potential exists to avoid the morbidity associated with anti-androgen therapy or with surgery for a significant proportion of patients. However, it should also be remembered that more than half of prostate cancer patients have cancers that harbour a fusion product, and we expect that many patients with clinically localised disease will be cured of cancer by prostatectomy, even if the fusion product is present.

Demichelis *et al* (2007) followed 111 patients undergoing watchful waiting for localised prostate cancer (Demichelis *et al*, 2007). Patients with the gene fusion had a 2.7-fold increase in prostate cancer-specific mortality, when compared with patients without fusion. However, 23% of 94 patients without the fusion protein experienced metastatic prostate cancer after 12 years of observation, when compared with 53% of 17 patients with fusion. Although the difference was statistically significant (*P*<0.01), a recurrence risk of 23% is not sufficiently low to endorse watchful waiting as an alternative to surgery. In a similar study, Attard *et al* (2008) studied 445 patients under watchful waiting. Fusion analysis was conducted on prostate specimens embedded in a tissue microarray using FISH. In this study, patients without the fusion transcript had a good survival experience (90% survival at 10 years). Attard *et al* (2008) refined their study by sub-dividing patients with fusion transcripts into three categories: (1) those with retained 5′ and 3′ *ERG* DNA sequences; (2) those with one retained copy of 3′ *ERG* sequence but no retained 5′ *ERG* sequence; and (3) those with two copies of the retained 3′ *ERG* sequence (i.e., homozygous or duplicated) but no retained 5′ *ERG* sequence. The third group was noteworthy for its poor prognosis; after 8 years of follow-up, patients in this group were six times more likely to die from prostate cancer than those with no *TMPRSS2:ERG* gene fusion (hazard ratio=6.1, 95% CI: 3.3–11.1, *P*<0.0001). Only 25% of patients in this class were alive at 8 years. The frequency of gene rearrangements in the Attard *et al* (2008) study was 30%, when compared with only 15% in the study by Demichelis *et al* (2007). It may be that the Demichelis group used a less sensitive assay to screen for the presence of the fusion gene, or that a number of variant transcripts went undetected, and that this resulted in misclassification and attenuation of the true effect (however, in a later study from the same group, the low prevalence of rearrangements was confirmed). The patients in the Demichelis study were from Sweden, and it is possible that the prevalence of the fusion protein varies with ethnic group. It remains to be proven that patients who lack fusion genes may be candidates for watchful waiting. Future studies might benefit from including additional markers of risk in the prognostic model, including grade, stage, PSA level, ethnic group, and the presence of one or more markers of genetic susceptibility, such as the recently defined cluster of markers on chromosome 8q24 (Amundadottir *et al*, 2006; Yeager *et al*, 2007; Zheng *et al*, 2007).

In a prospective cohort study, Nam *et al* (2007b) examined the effect of the most common fusion variant (T1/E4) on disease recurrence (defined by a rising PSA level after surgery) among 165 patients who underwent surgery for localised prostate cancer (Nam *et al*, 2007b). This particular gene fusion was present in 49% of patients, and 26% of the patients developed biochemical disease recurrence. Patients with fusion had a much higher rate of recurrence (54% at 5 years) than those without fusion (8%). Fusion status did not correlate with PSA, grade, or stage. After adjusting for PSA, grade, and stage, the hazard ratio for recurrence was 8.5 (95% CI: 3.6–20.6, *P*<0.0001). This study implies that this biomarker may be an independent prognostic factor of disease recurrence. However, although all patients who die of prostate cancer experience biochemical recurrence before death, only a minority of patients with biochemical recurrence succumb to prostate cancer. It is important that large studies of prostatectomy patients be conducted with longer follow-up and that additional end points include distal recurrence and prostate-specific mortality. The T1/E4 variant is the most common of *TMPRSS2:ERG* gene fusions and is the best studied; it is not yet known if other variants are associated with prostate cancer prognosis to the same extent. Furthermore, it is not clear why the results of the three cohort studies (which examined the presence of the fusion protein) had results that differed dramatically from those of Petrovics *et al* (2005) who examined the overexpression of ERG. Petrovics *et al* measured overexpression in cancer tissues relative to benign prostate, and it is possible that expression levels in the surrounding stroma are clinically relevant as well. In an ideal study, one would perform both assays on a single group of patients.

The *TMPRSS2:ERG* gene fusion is specific for prostate cancer, and the ability to identify this DNA rearrangement could be used as a screening test for prostate cancer in serum, prostatic fluid, or in urine. One study has been conducted on DNA specimens isolated from urine from men known to have prostate cancer with a gene rearrangement (Hessels *et al*, 2007). The sensitivity of the urine test was only 37% and the specificity was 93%. It is possible that future assays will have comparatively better sensitivity or that the presence of the fusion gene in urine could supplement a panel of markers in a screening setting.

## Conclusions

In conclusion, the discovery by Tomlins *et al* in 2005 of a frequent genetic rearrangement in prostate cancers has changed our conception about the role of chromosomal rearrangements in the aetiologies of common solid tumours. In the short time since this discovery, several authors have confirmed the importance of this genetic fusion, and have expanded the class of fusion genes greatly. It now appears that these are among the most frequent recurrent rearrangements in cancer. The consequence of the various fusion transcripts is the overexpression of a member of the *ETS* family of oncogenes, initially under the control of androgen and the androgen receptor, but androgen dependence may be lost in advanced disease. It now appears that activation of this pathway may be central to prostate carcinogenesis, but the clinical implication of the various fusion products has not been worked out. It is hoped that this discovery will quickly lead to treatments tailored to patients in different risk classes, and possibly to a screening test, and ultimately it is hoped that the *ETS* family oncogenes will be molecular targets for novel therapies.
